# Leading matters! Linking compassion and mindfulness in organizations through servant leadership

**DOI:** 10.3389/fpsyg.2024.1346751

**Published:** 2024-04-08

**Authors:** Sandra Miralles, Anne B. Pessi, Manuela Pozo-Hidalgo, Alma Rodríguez-Sánchez

**Affiliations:** ^1^Department of Management and Marketing, Universitat Jaume I, Castellón, Spain; ^2^Faculty of Theology, University of Helsinki, Helsinki, Finland; ^3^Business Area, Faculty of Social Science and Law, Valencian International University, Valencia, Spain

**Keywords:** compassion, mindfulness, servant leadership, safety, organizational well-being

## Abstract

Regardless of where they are, humans are inherently human. In this study, we explore the relationship between compassion, mindfulness, and servant leadership contributing to an overall feeling of safety. Adopting a humanistic approach to human resource management, we examine how compassion and mindfulness intersect under the lens of the Conservation of Resources (COR) theory. Our investigation focuses on understanding how servant leadership facilitates the cultivation of mindfulness through compassion. Using structural equation modeling (SEM), we analyze data gathered from 360 workers across diverse occupational sectors. Our findings provide empirical support for the hypothesis that compassion, manifested as a response to suffering, enhances mindfulness levels in the workplace. Specifically, we observe that organizations promoting servant leadership principles are conducive to higher levels of mindfulness among employees. Practically, our study underscores the importance of designing work contexts that prioritize compassion and servant leadership. By doing so, organizations can foster a positive work environment that promotes mindfulness and enhances workplace safety. Our research contributes to the management literature by offering empirical evidence on the role of servant leadership in cultivating compassion and mindfulness, thereby advancing the discourse on workplace safety and organizational well-being.

## 1 Introduction

Humans are human everywhere. Despite the fact that emotions and feelings emerge in all workplaces, it is not always easy to incorporate humanistic view for managing organizations, particularly in highly competitive, hectic contexts. There is an increasing trend focused on the study of the human side of management, where feelings and emotions matter (Çakıt et al., 2020; Pessi et al., 2022; Zhang et al., 2022; Ramachandran et al., 2023; Zhang et al., 2023). However, further research is urgently called for; We lack academic work understanding not only on emotions in organizations but also the ways in which emotions are part of our embeddedness in the social world (Zhang et al., 2023), part of our existence, also at work.

Classically, emotions have been explored in the level of individual, as intra-individual processes, much less on interaction and communities such as workplaces. Recent work has emphasized the opposite: emotions in organizational processes and learning (Maitlis et al., 2013; Weick and Sutcliffe, 2015; Mikkelsen et al., 2020), emotional culture of organizations (Barsade and O’Neill, 2016), and, for instance, the contagiousness of emotions (Pugh, 2001). We wish to balance between the two approaches by bringing more individuals-related phenomenon of mindfulness into dialog with elementally social compassion. In particular, we are inspired by the question whether compassion has the potential to help people become more focused on the present, and more aware of experiences and emotions without judgment, namely, more mindful (Brown and Ryan, 2003).

Research illustrates that mindfulness is beneficial for employees because it helps them to respond to work challenges without reacting in an impulsive or preoccupied manner (Good et al., 2016; Yu and Zellmer-Bruhn, 2018). Through mindfulness, workers can develop their self-regulatory capacities (Glomb et al., 2011), enabling them to manage and understand their emotions better in challenging situations at work, and actually to benefit from emotions, promoting also their sense of safety. Effectively managing emotions can impact the sense of security by enabling individuals to adapt more positively to challenges and stressors. Furthermore, mindfulness practice is linked to a significant decrease in stress and anxiety levels (Carmody and Baer, 2008). Chronic stress can negatively impact the sense of security, and mindfulness’s ability to reduce stress responses may contribute to an overall feeling of safety.

There is little empirical research on the factors that affect mindfulness in the workplace (Shahbaz and Parker, 2022). Thus, it is important to understand and explain the mechanisms through which mindfulness is produced and maintained in the workplace, as well as the mediating factors that enhance it. This is crucially needed in order to improve the application of research in professional practice and to develop more humanistic, safe emotions-understanding, mindful organizations. Individual and workplace factors can promote employees’ mindfulness experiences even without mindfulness interventions (Hülsheger et al., 2018). Compassion practices in the everyday of organizations can be one such factor, being a highly effective healing and safety-promoting tool, capable of increasing workers’ mindfulness levels by facilitating the ability to focus on the present moment (Shao and Skarlicki, 2009).

Compassion with one another is the cornerstones of human sense of safety. Compassionate environments contribute to alleviating the suffering of others, and when compassion is welcomed, valued and expressed in an organization, both by employees and leaders, the right conditions are created for people to experience a state of mindfulness, enabling sense of safety. There is a need to better understand the contextual factors affecting the process of emergence of mindfulness. In this vein, we posit that leaders should help employees both emotionally and cognitively to deal with organizational challenges (Obi et al., 2020); They may catalyze the impact of employees’ compassion on their mindfulness. In other words, there is a mutual synergy between mutual, interactional compassion and individual mindfulness.

But how is such a synergy promoted and mediated? We claim that servant leadership may have particular potential as a mediator in this relationship. Servant leaders prioritize the individual needs and interests of workers, promoting their development through altruistic and ethical behaviors (Greenleaf, 1977). In addition, they are able to generate environments in which employees feel safe, through behaviors such as compassion, enthusiasm, and empathy, increasing employees’ sense of psychological safety (Ma et al., 2021).

Therefore, the objective of this study is to explore the ways in which servant leadership exerts a mediating role in the relationship between compassion and mindfulness, toward (among other positive phenomena) sense of safety at work. As part of this focus, we will also confirm if compassion positively affects servant leadership, and if servant leadership positively impacts employees’ mindfulness. This study, thus, aims to advance knowledge of the mechanisms by which compassion is related to mindfulness at work, enhanced by the effect of servant leadership as a mediating variable, to better understand the factors that facilitate mindfulness at work. This research is supported by the umbrella of the conservation of resources theory (COR), according to which people orient their behavior toward the protection of their current resources, and the acquisition of new ones, understanding resources as states or conditions that people value, and try to maintain, such as compassion or servant leadership (Hobfoll, 1989, 2011).

To this end, this paper is structured in four sections. The first section presents a theoretical description of the hypotheses of the research model. The second section describes the methodology, including the sample, instruments and analyses carried out. This is followed by the results of the study, and finally, the conclusions, implications and future lines of research are presented.

## 2 The relationship between compassion and servant leadership

Employees in an organization may be undergoing unpleasant subjective experiences like psychological distress, physical or emotional pain, or existential anguish. This suffering of employees in their workplace can come from experiences in their private lives or from specific circumstances to their job or their organization. In any case, employee suffering knows no barriers (Lilius et al., 2011). Consequently, this anguish can markedly impact employees’ sense of safety in the workplace.

The phenomenon is two-fold. On the one hand, personal suffering outside of work itself could result from a personal setback like loss or serious illness of a loved one (Lilius et al., 2008), chronic pain or physical/psychological illness (Dewa and Lin, 2000), the breakup of a romantic relationship (Manns and Little, 2010), natural disasters such as earthquakes, floods or man-made disasters such as fires or terrorist attacks. On the other hand, suffering generated in the workplace can come from many sources, e.g., through organizational stressors such as layoffs, workplace injuries, restructuring, or incivility from supervisors and colleagues (Frost, 2003; Driver, 2007).

An antidote to both kind of suffering might be compassion. Compassion can be defined as an interpersonal process involving the noticing, feeling, sensemaking and acting that alleviates the suffering of another person (Dutton et al., 2014). Specifically, compassion is a process that involve four phases: (1) noticing the suffering of others, (2) making meaning of suffering in a way that contributes to a desire to alleviate it, (3) feeling empathic concern for these people, and (4) acting to eliminate or alleviate their discomfort (Worline and Dutton, 2017). Moreover, according to Dutton et al. (2014, p. 283), “acting compassionately can involve a breadth of different behaviors, ranging from mere presence or listening to more elaborated, coordinated, and durable actions that involve directing multiple resources toward a sufferer.”

In this sense, compassion, rooted in caring and a willingness to help others, aligns with the concept of prioritizing employee needs in servant leadership (Liden et al., 2008) since servant leaders are deeply impacted by the problems around their followers. Additionally, Graham (1991) describes servant leadership as a style that integrates principles, ethics, and integrity. Servant leadership focuses on followers, prioritizes their needs and interests, and involves a shift in concern from self to others within the organization and society as a whole (Eva et al., 2019). A servant leader listens to and understands his followers’ main goals and coaches them to achieve them. Therefore, servant leaders focus on the growth of those they lead.

Therefore, what distinguishes servant leadership is its emphasis on helping followers reach their full potential and meet the requirements of broader stakeholder groups. To ensure that employees exploit their potential, it is essential that suffering is not present in their lives, or at least that it does not limit them excessively. Consequently, alleviating employee distress and suffering becomes imperative for a servant organizational leader constituting a fundamental aspect of its guiding principles.

According to van Dierendonck and Patterson (2015), compassion can motivate servant leaders to be more empathetic and better address the suffering of others as it makes it easier to notice the employee’s suffering. Compassion triggers the need to understand the situation (perspective taking) and take action to alleviate the employee’s suffering (compassionate response). In other words, the leader’s compassion toward the follower represents a dyadic process in which the leader notices the follower’s suffering, feels empathic concern, makes sense of it, and responds with compassion (Miller, 2007; Lilius et al., 2008; Margolis and Molinsky, 2008). Actually, moreover, some researchers define servant leadership to include mutual compassion, and particularly the compassionate mindset by the leaders and directors (Pessi and Hakanen, 2018; Paakkanen et al., 2020).

To its core, servant leadership is characterized by several key attributes: (1) Helping subordinates grow and thrive: the leader is committed to their employees’ professional growth as well as their well-being by offering quality assistance and guidance. (2) Putting subordinates first: the leader sacrifices personal interests and prioritizes the needs and expectations of their followers. (3) Emotional healing: The leader cares about the problems and feelings of others and can restore the emotional well-being of followers from their emotional healing orientation. Therefore, to be a good servant leader they must have high levels of compassion. They alleviate the suffering of followers to ensure their mental health, empower them and help them grow professionally and personally (Wheeler, 2011). Barbuto and Wheeler (2006) referred to emotional healing as the ability of leaders to facilitate the recovery of sufferers after traumatic episodes. Servant leaders can facilitate this recovery by being compassionate and showing empathy for their problems (Liden et al., 2008). Additionally, leaders who are perceived by their colleagues as capable and willing to take responsibility for their emotional healing foster strong, positive relationships with their followers.

Therefore, the unique orientation of a servant leader for emotional healing and promotion of sense of safety may be strongly linked to his/her characteristic of listening, empathy, and compassion. Overall, the philosophy of servant leadership revolves around a willingness to help others, understanding and prioritizing the needs of followers. Therefore, compassion can act as a catalyst for a servant leader to help employees overcome their distress, alleviate pain and restore their emotional balance by fostering a compassionate approach.

In conclusion, a servant leader’s clear orientation toward emotional healing may be strongly linked to his or her ability to experience compassion. Certainly, the philosophy of servant leadership revolves around compassion, empathy, and prioritizing the needs of followers. Therefore, servant leaders, through their compassionate approach, could help their employees overcome their distress and restore their emotional balance. Based on the argument offered, the first hypothesis can be proposed:

*H1*. Compassion is positively and significantly related to servant leadership.

## 3 The relationship between servant leadership and mindfulness

Servant leadership represents a moral form of leadership that has experienced a great deal of interest in recent years (Lemoine et al., 2019). This is due, on the one hand, to the growing need for more moral and ethical leadership styles, on the other hand, to the potential success it can impact on organizations (Harrison, 2018).

For servant leaders, the priority is to meet the personal needs and interests of employees, development through altruistic and ethical behavior (Greenleaf, 1977). Based on the definition recently proposed by Eva et al. (2019), servant leadership is an (1) other oriented approach to leadership (2) manifested through one-on-one prioritizing of follower individual needs and interests, (3) and outward reorienting of their concern for self toward concern for others within the organization and the larger community’ (Greenleaf, 1977).

This definition captures three key characteristics that differentiate this and other approaches to leadership, namely motive, mode and mindset. Motive refers to leadership focused on others, with a personal motivation to serve in an altruistic way. Mode refers to the individualized prioritization of the individual needs, interests and goals of his or her collaborators over those of the leader himself or herself. Finally, mindset involves a focus on the development of their collaborators and a concern for the wider community and a commitment to being responsible for their well-being (Eva et al., 2019).

Research has shown that servant leaders exhibit a strong development orientation, provide appropriate feedback to their teams and support skill development (Chen et al., 2015). In addition, they excel in qualities such as empathy, healing, conscientiousness, persuasiveness, stewardship and commitment to employee growth (Sherman, 2019). When employees perceive servant leaders’ interpersonal behaviors, such as compassion, enthusiasm, and empathy, it creates an environment in which they feel more comfortable expressing their thoughts and opinions, increasing employees’ sense of psychological safety (Ma et al., 2021).

Servant leaders develop an atmosphere of psychological safety, through support, trust and open communication (Zhao et al., 2020). They show sensitivity, consideration and concern for their employees (Andersen, 2018). This attitude of genuine concern and care for the well-being of their employees can have a significant impact on teams’ level of awareness as they feel safe, valued and understood.

In the last decades, there has been a growing interest in mindfulness in different research fields, due to positive findings on the relationships between mindfulness with job performance (Dane, 2011) and employee well-being (Glomb et al., 2011). In addition, numerous studies also showed this relationship with positive attitudes and behaviors in organizational settings (Dane and Brummel, 2014; Reb et al., 2015), such as self-regulation, which allows people to take greater control over their actions and emotions, reducing their impulsivity (Glomb et al., 2011).

Although researchers have not yet reached a consensus on the specific definition of mindfulness, we note some agreement in defining it as a state of awareness in which the person focuses attention on the events and experiences of the present moment (Brown and Ryan, 2003). That is, mindfulness is the process of paying attention to what is happening in the present moment, both to internal stimuli, such as thoughts or sensations, and external stimuli, the physical and social environment, and observing these stimuli without prejudice.

However, empirical research on mindfulness at work is limited. Furthermore, there is little knowledge about what motivates employees to engage in mindfulness, as research has so far focused on the practice of meditation and its outcomes (Reb et al., 2015). These authors found high correlations between leaders support and employees’ level of mindfulness, suggesting the important role that leaders, for instance supporting leaders, play in encouraging or limiting mindfulness at work, positing that mindfulness can not only be enhanced through personal practice or meditation, but also through other organizational or situational factors.

Employees who perceive that the organization cares about their development and well-being tend to be more mindful than others. Therefore, leadership style and its behavior toward employees is one of the organizational factors that enhances mindfulness.

We argue that servant leadership could generate the right conditions for fostering employees’ mindfulness. This particular leadership style focuses on prioritizing its employees’ goals over its own, making employees feel confident that it will put their well-being before organizational goals (Dirks and Ferrin, 2002). Furthermore, servant leadership emphasizes support for employees through psychosocial needs-oriented behaviors and promotes employee well-being, which has been postulated as a key mechanism to explain the positive relationship of leadership on mindfulness (Gui et al., 2021). Hence, leaders, specifically servant leaders may be perceived by the employees as a significant resource (Hobfoll, 2011).

Moreover, servant leaders develop a climate in which workers feel valued and listened to, creating a safe psychological climate (Van Dierendonck, 2011). Other authors (Kalafatoğlu and Turgut, 2019) observed in their study that individual goal orientation and supportive organizational climate predict a higher level of mindfulness in employees. Therefore, those who focus on self-development and perceive that the organization cares about their well-being tend to be more mindful than others.

Likewise, when relationships between leaders and their employees are positive and of high quality, it generates positive outcomes through employees’ positive attitudes in the work environment, showing better performance at work (Graen and Cashman, 1975).

Therefore, we posit that in teams where there is an internal willingness of leaders to care about others, there is a greater likelihood that workers will develop higher levels of mindfulness.

*H2*: Servant leadership is positively and significantly related to mindfulness.

## 4 The mediating role of servant leadership in the relationship between compassion and mindfulness

Fundamentally, there is—under-studied—synergy between compassion and being mindful; A mindful person has a propensity to be open to novelty, attentive to distinctions, sensitive to context, aware of multiple perspectives, and focused on the present (Bodner and Langer, 2001). Compassionate people, who feel, care, and act to alleviate the suffering of others, can create the foundation for being in a state of mindfulness. The practice of mindfulness allows individuals to look deep down into their hearts, question their own views and beliefs, think more critically, and recognize all the wisdom they have within themselves. However, suffering can prevent people from being in a state of mindfulness, as psychological distress, physical or emotional pain, or existential anguish make the principles of mindfulness difficult. Therefore, compassion within organizations turns out to be a very powerful tool for healing that can increase the levels of mindfulness of their workers, helping them focus on the here and now (Shao and Skarlicki, 2009).

According to resource conservation theory, people strive to protect, conserve, nurture and obtain more of the valuable resources essential to their well-being. Therefore, by obtaining more resources, we enable people to reduce their stress levels by being better able to cope with threatening situations (Hobfoll, 1989). We suggest that experiences of compassion can elicit positive emotions about people in the organization, generating resources related to improving mindfulness such as self-efficacy, resilience, optimism and hope (Ko and Choi, 2019). Through these resources, a person can interpret a certain unfortunate event in a more positive way, which helps them to focus on the present and avoid negative feelings and thoughts. According to this view, as people experience compassion at work, they also develop new resources to enhance related positive behaviors, such as mindfulness. Furthermore, compassion experienced in the context of an organization triggers positive emotions among its workers, contributing to the development of positive psychological states among members of the organization (Lilius et al., 2008).

Therefore, when compassion has a place and acceptance in an organization, the ideal circumstances are created so that people can be in a state of mindfulness. That is, fostering compassion among employees and leaders can create a positive work environment in which mindfulness can flourish. In this sense, the effects of mindfulness will be enhanced to the extent that there is a clear and shared purpose, a good fit of values between members and the organization in general, and a compassionate attitude (Rupprecht et al., 2019).

Furthermore, to achieve higher levels of mindfulness it is necessary to listen deeply. For Armstrong (2011), this deep and active listening that can allow us to let go of the ego involves the practice of mindfulness. For its part, compassion generates a work environment that is perceived as supportive and supportive (Prieto and Pérez-Santana, 2014). Specifically, in a work environment where compassion predominates, people trust each other and tend to feel psychologically safe to discuss problems and issues openly, combine forces, and therefore actively listen to their colleagues. On the other hand, compassion helps people face mistakes and failures with an open mind and heart (Edmondson, 2012). All of this makes it easier to carry out mindfulness. Therefore, compassion facilitates mindfulness because it creates conditions for altruism and intrinsic motivation, for taking risks, for talking about mistakes, worries and problems, for developing better ways of doing things and for creating a climate of optimism, effectiveness and cohesion in teams (West et al., 2017), thereby facilitating the practice of mindfulness.

Finally, compassionate environments would be more conducive to facilitating information exchange, since solving others’ problems and helping to alleviate their suffering is the essence of compassion. In summary, compassion can stimulate mindfulness, as a compassionate work environment increases the degree to which employees believe that their workplace provides the interpersonal support necessary to feel free to experience mindfulness. Specifically, Atkins and Parker (2012) relate compassion to mindfulness in four points that can define mindfulness: (1) Contact with the present moment; (2) Defusion of thoughts and feelings; (3) adopting an approach to observe oneself; (4) Acceptance of negative thoughts and feelings.

Servant leadership is a style that stands out as being particularly suited to enhance mindfulness, as it stands out for prioritizing the needs of its followers and fostering a supportive work environment. In this way, servant leadership will have a mediating effect on employees’ compassion and mindfulness. Previous research has found evidence that individual and organizational factors can promote experiences that help increase mindfulness at work (Hülsheger et al., 2018). Knowing what these factors are would enable organizations to move forward in implementing the most effective models for employee management practice (Shahbaz and Parker, 2022). Servant leadership could be one of those decisive factors in the organizational environment, that can be perceived as a crucial resource or a resource provider by the employees (Hobfoll, 2011).

On the one hand, compassion can help servant leaders to be more empathetic and contribute to alleviating the suffering of others. These leaders are humble, respectful, and appreciative of their employees’ differences, and this helps to develop an open dialog through listening (Van Dierendonck and Patterson, 2015). In addition, these shows of support from leaders toward employees have a great influence on the level of employee awareness (Reb et al., 2015). On the other hand, compared to other leadership styles, servant leadership might help to create a safer emotional environment for employees (Schaubroeck et al., 2011) due to its focus on acceptance, which is a key element for employees to develop mindfulness. Furthermore, compassion and mindfulness are closely related to emotional well-being, and this leadership style shows more concern for employees’ emotional well-being than other leadership theories (Sendjaya, 2015; Newman et al., 2017).

Considering the above, this study proposes that servant leadership may be affected by employees’ compassion, and in turn enhance mindfulness. When employees exhibit caring behaviors through organizational environments that promote supportive leadership styles toward workers, their levels of mindfulness are likely to increase.

Thus, we propose:

*H3*: Servant leadership mediates the relationship between compassion and mindfulness.

## 5 Research methodology

### 5.1 Organizational context

The study was conducted among Spanish companies engaged in various activities, including manufacturing, construction, and services such as education, healthcare, and finance. This approach provides a comprehensive view of the diverse economic landscape under examination.

The required size of companies is small business and upwards. That is, no data was collected from companies with fewer than 10 workers (micro-companies). Specifically, the final sample is made up of 51% small companies, 37% medium-sized companies and 12% large companies.

Various employees from each company were surveyed, and it was mandatory for participants to have a minimum of 6 months of employment with the company. This timeframe is considered reasonable, ensuring that the employees have gained a thorough understanding of their workplace.

### 5.2 Participants and procedure

We formed a stratified sample of 360 workers from companies in different occupational sectors. Data collection within each company was conducted through questionnaires that were answered through telephone calls, covering all the items that required responses from the workers. Researchers were giving the informed consent to the different companies’ participants before data gathering. The number of responses exceeded the minimum threshold of 100 subjects necessary for the application of structural equation methodology, and to be able to test the psychometric properties of the measurement scales (Spector, 1992; Williams et al., 2004). All items were measured on a 7-point Likert scale. All indicators on the Likert scale were expressed positively except for the mindfulness scale which was measured negatively. All respondents had to indicate whether they agreed or disagreed with each statement included in the questionnaire.

### 5.3 Measurements of the variables

We used the original measurement scales, except for the servant leadership measurement scales, in which we maintained the same items but we tailored it to the follower’s perspective.

Mindfulness was measured by four items adapted from MAAS: Brown and Ryan (2003) which is a conspicuous scale of the western approach on mindfulness. An example item is: “It seems to me that I work automatically without paying much attention to what I do.”

Compassion was measured with the four-item compassion scale adapted by Petchsawanga and Duchon (2009), which follows the Dutton et al.’s (2014) perspective, selected in this research. An example item is: “I easily put myself in the shoes of others.”

Servant leadership was measured through seven items from the Liden et al. (2015) scale, which was adapted from the leader’s version to show the opinion of team members. An example item is “for him/her, my personal development is a priority.” To measure this scale, workers had to respond by thinking about their team leader.

Control variables. Age (in years) and gender was used as a control variable. We controlled for these variables because they have been shown to influence compassion as well as similar constructs (Lilius et al., 2008).

### 5.4 Data analysis

Firstly, we obtained descriptive analyses, intercorrelations and reliabilities (Cronbach’s alpha) using SPSS (28.0.0.1, 14). Second, we conducted Harman’s single-factor test (Podsakoff and Organ, 1986) to assess whether common method variance existed and to address potential social desirability bias in the responses. This test is convenient to perform when subjective evaluation measures are used. SPSS was used to check common method bias. If the total variance extracted by one factor exceeds 50%, it means that common method bias is present in our model. The results reported that the total variance extracted by one factor is 23.45%, and it is below the recommended threshold of 50%, thus confirming no issues of common method bias. Third, we performed the analysis of the measurement model and the structural model by means of Process (v. 4.2) macro by Hayes’s (2013) in SPSS.

## 6 Results

First, Table 1 shows the descriptive statistics, alpha coefficients and factor correlations of the study variables. Two items from the servant leadership measurement scale were removed as they presented low factor loadings. The Cronbach’s alpha coefficients range from 0.6 to 0.7, which is below the minimum accepted value of 0.7, as recommended by Nunnally and Bernstein (1994). Nevertheless, it is important to note that reliability standards can vary based on the context and purpose of the instrument. In this study, the instrument’s reliability was evaluated using Cronbach’s alpha measurement, with an emphasis on internal consistency. According to established criteria, an alpha value greater than 0.6 is considered indicative of reliability, with acceptability in the range of 0.6–0.8 (Hajjar, 2018). Given these criteria, the slightly lower alpha values observed in our study can still be considered acceptable within the specified reliability framework, especially when considering the flexibility and more subjective measures inherent in social science research.

**Table 1:** Means, standard deviations, alpha coefficients and correlation coefficients for test variables.

		Mean	SD	Compassion	Servant	Mindfulness
1	Compassion	6.358	0.458	(0.680)		
2	Servant leadership	5.469	0.624	0.122*	(0.720)	
3	Mindfulness	6.393	0.478	1.173**	0.247**	(0.680)

*N* = 360. Cronbach’s alpha coefficient is reported on the diagonal, in parentheses. **Statistically significant correlation coefficient (*p* < 0.01) (two-tailed). *Statistically significant correlation coefficient (*p* < 0.05) (two-tailed).

The results of Harman’s single factor test showed a poor fit: [Chi square (df) = 657.048 (90); *p* < 0.01; BBNFI = 0.429; TLI = 0.367; CFI = 0.457; RMSEA = 0.173]. Consequently, and in accordance with this procedure, we do not consider common method variance to be a problem in our research.

Then, we checked the measurement model by running confirmatory factor analyses (CFA) using the Lavaan “R” package (Rosseel, 2012). We used Tucker-Lewis Index (TLI), Comparative Fit Index (CFI), Root mean square error of approximation (RMSEA), and standardized root mean squared residual (SRMR). CFI and TLI are incremental fit indices used to assess the improvement in fit of a hypothesized model compared to a baseline model. RMSEA is classified as an absolute fit index because it evaluates the deviation of a hypothesized model from an ideal model. SRMR is a precise indicator of model adequacy that checks the differences between the observed correlation matrix and the correlation matrix predicted by the model. Values greater than 0.90 TLI and CFI (Hoyle and Panter, 1995), and smaller than 0.08 for RMSEA (Bentler, 1990) and indicate an acceptable fit. SRMR values falling within the range of 0.1 to 0.08 indicate congruence between the data and the model (Henseler et al., 2014). The results confirm an adequate fit of the model with the data used (TLI = 0.903; CFI = 0.919; RMSEA = 0.073; SRMR = 0.069).

Table 2 shows average variance extracted (AVE) and composite reliability values (CR). The AVE ranges between 32 and 58%, below the recommended value of 0.5. Following Fornell and Larcker (1981), the AVE could present a more cautious evaluation of the measurement model’s validity, suggesting that solely relying on composite reliability might lead the researcher to consider the convergent validity of the construct as satisfactory. Given that CR values are above the recommended threshold 0.6 (Fornell and Larcker, 1981), we can state that the internal reliability of the measures is acceptable.

**Table 2:** Average variance extracted and composite reliability values.

	AVE	CR
Compassion	0.583	0.842
Servant leadership	0.386	0.785
Mindfulness	0.320	0.695

AVE, Average variance extracted; CR, Composite reliability.

Second, we evaluated the structural model. Hypothesis 1 predicted a positive and direct effect of compassion on servant leadership. Results for the regression coefficients of the model indicate a positive and direct relationship between compassion and servant leadership (β = 0.216; *t* = 2.301; *p* = 0.023; LLCI = 0.030; UCLI = 0.401). Hypothesis 2 predicted a positive and direct effect of servant leadership on mindfulness, and the results confirmed this relationship (β = 0.402; *t* = 5.114; *p* < 0.01; LLCI = 0.246; UCLI = 0.557). Table 2 shows the direct effects results.

**Figure 1:**
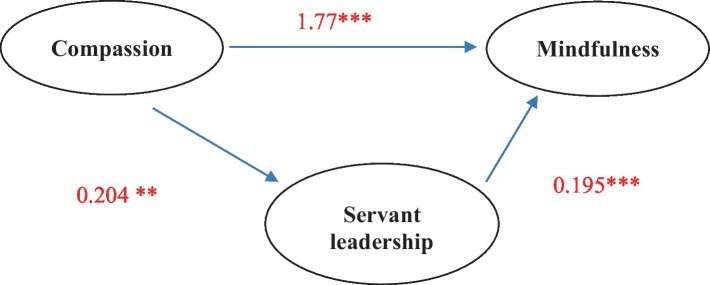
Mediation model. **Significant correlation (*p* < 0.01), ***Significant correlation (*p* < 0.001).

Hypothesis 3 predicted a mediating effect of servant leadership in the relationship between compassion and mindfulness. The estimated indirect effect of compassion on mindfulness via servant leadership is 0.077. The 95% bias-corrected confidence interval for the indirect effect (ab) based on 5,000 bootstrap samples was entirely above zero (0.003–0.174). Thus, the indirect effect of compassion on mindfulness is significantly different from zero and the null hypothesis of no mediation effect can be rejected. Therefore, Hypothesis 3 is also confirmed (see Table 3). In other words, the relationship between compassion and mindfulness occurs through servant leadership (Figure 1). Regarding the control variables, only age revealed a significant effect.

**Table 3:** Compassion, servant leadership and mindfulness.

Models and variables	*B*	SE	*t*	*p*	95% CI	*R* ^2^_adjusted_
Model on servant leadership						0.023*
Compassion	0.204**	0.071	2.868	0.004		
Model on mindfulness						0.107***
Compassion (H1)	0.177***	0.053	3.353	<0.001		
Servant leadership	0.195***	0.039	5.047	<0.001		
Total effect model on mindfulness						0.043***
Compassion	0.217***	0.054	4.021	<0.001		
Indirect effect (H2)						
Servant leadership	0.04	*(Boot)* 0.023			0.006/0.096	

*N* = 132. *p < 0.05; **p < 0.01; ***p < 0.001.

## 7 Discussion

The contribution of this paper is three fold. First, we adopt a humanistic approach for managing human resources, and bring to life the concept of mindfulness in a managerial context. Second, we propose a connection between the humane capacity of compassion and mindfulness, under the approach of the COR theory. Third, we integrate in a novel way compassion, leadership, and mindfulness, in such a way as to confirm a potential mediating role of servant leadership in the relationship between compassion and mindfulness.

This research corroborates how organizations increasingly focus on the human side of management and look for leaders who can lead the organization by adopting a leadership style that fosters a sense of safety in organizations. Servant leaders, with their service orientation toward others and their pro-follower management approach, seem to fit this framework.

The study demonstrates how servant leaders, with a compassionate orientation toward employee suffering, can play a crucial role in creating an organizational environment in which employees can experience mindfulness. These findings align with previous studies showing the positive link between servant leadership and compassion (Ahmad et al., 2023).

Therefore, servant leaders, who show concern for their followers, ensure that compassionate people can act genuinely as they are, without faking and thus reach high levels of mindfulness, which can improve their performance (Miralles-Armenteros et al., 2021). In this regard, organizations must prioritize the development of servant leadership and foster a culture of compassion to promote positive states in their workers such as experiencing mindfulness, and then sense of safety too.

Continuing with the argument, more compassionate people will have more tools to exercise servant leadership that is able to understand the suffering of others and, because of that understanding, begin to work with the sufferer so that they can emerge from their discomfort. Thanks to this, there will be more chances of finding an environment in which mindfulness is possible.

Therefore, the servant leaders are willing to help their followers to alleviate or eliminate their suffering. While there are many tools available to achieve this, compassionate action appears to be the most appropriate response to the world’s suffering (Davenport, 2015). In line with this, leaders support would be a fundamental aspect to enhance mindfulness in compassionate organizational environments. In fact, those employees who perceive greater organizational support provided by their leaders will have the resources to be more mindful (Reb et al., 2015), highlighting the relevant role of the leader in this relationship. Despite the importance of the leader’s role in the development of mindful organizations, research on mindfulness in the field of leadership is still scarce (Verdofer, 2016).

### 7.1 Implications

The insights provided by this study into the servant leader’s approach to emotional healing will guide leaders in organizations to understand and practice the process of alleviating employee suffering so that a culture of compassion and mindfulness emerges and is sustained creating a sense of security in the organization. This approach goes in line with the call in the literature to humanizing workplaces and organizations (Black and Venture, 2017; Pessi et al., 2022).

On the other hand, employees who perceive that their leaders exercise a servant leadership style, in which they show concern and interest in their needs and development, tend to be more mindful, and more focused on the present. Our findings confirm that the perception of leaders’ support has a positive effect on employees, being one of the organizational aspects that strengthen mindfulness enhancement.

Leaders who adopt this approach inspire their employees to work with greater compassion, empathy and to develop a greater capacity for mindfulness at work, contributing significantly to enriching a positive work environment where mindfulness progresses.

Finally, the present study also contributes to the advancement of the almost nonexistent empirical literature on the role of leadership in emotional healing, compassion and the state of mindfulness.

From a practical application point of view, this study highlights the need for organizations to train leaders to develop more humane behavior that truly puts people in a relevant position. Through a servant leadership orientation, leaders acquire the skills necessary to understand and meet the needs of their team, developing empathy, active listening and showing concern for their well-being. These attitudes create an environment of trust and confidence, which increases their employees’ ability to be present and focused through their support, thus generating positive results for both individuals and organizations. This is crucial for promotion of sense of safety in all workplaces.

### 7.2 Limitations and future research

It is important to note that the study has limitations, such as its cross-sectional design. Longitudinal and experimental studies can provide further insights into the causal effects and mechanisms underlying this relationship.

There is a need to further explore the connection among compassion, servant-leadership and mindfulness, as this discussion is only the beginning of the examination. If compassion is the appropriate response for servant leaders, there is a strong need to explore how compassion can be implemented and developed in organizations, and to promote mindfulness in employees.

Therefore, future research can continue to examine the effects of other leadership styles on mindfulness capacity in organizations. The correlations between servant leadership and mindfulness, as well as the limited previous literature on leader behaviors and mindfulness levels, open the opportunity to further explore this relationship.

## 8 Conclusion

In this study we have demonstrated the crucial role that servant leadership plays in improving workers well-being, in this case in form of mindfulness. Servant leadership truly seems to have the power to promote the synergy between compassion and being mindful. This leadership style stands out for its concern for people, where leaders are able to create a work environment in which employees feel safe, listened to, developed and cared for. In this way, they manage to generate compassionate environments that help alleviate the suffering of others, with compassion being a fundamental factor in improving employees’ levels of mindfulness capacity by facilitating their ability to be more aware of experiences and emotions without judgment. Compassion in organizations is absolutely needed, but if leaders do not follow the care principles toward their employees, i.e., concern for their needs and interests in a genuine way, the power of compassion dissipates. So also, mindfulness is a decisive aspect for employees, because it facilitates their abilities to cope with work challenges, and enhances their emotional regulation capacities, which allows them to increase their sense of security.

## Data availability statement

The raw data supporting the conclusions of this article will be made available by the authors, without undue reservation.

## Ethics statement

Ethical review and approval was not required for the study on human participants in accordance with the local legislation and institutional requirements. Written informed consent from the patients/ participants or patients/participants’ legal guardian/next of kin was not required to participate in this study in accordance with the national legislation and the institutional requirements.

## Author contributions

SM: Writing – original draft. AP: Writing – original draft. MP-H: Writing – original draft. AR-S: Writing – original draft.
