# Corrigendum: Leading matters! Linking compassion and mindfulness in organizations through servant leadership

**DOI:** 10.3389/fpsyg.2024.1513605

**Published:** 2024-10-30

**Authors:** Sandra Miralles, Anne B. Pessi, Manuela Pozo-Hidalgo, Alma Rodríguez-Sánchez

**Affiliations:** ^1^Department of Management and Marketing, Universitat Jaume I, Castellón, Spain; ^2^Faculty of Theology, University of Helsinki, Helsinki, Finland; ^3^Business Area, Faculty of Social Science and Law, Valencian International University, Valencia, Spain

**Keywords:** compassion, mindfulness, servant leadership, safety, organizational well-being

In the published article, there was an error in the author list. Anne B. Pessi should be listed as the second author and as a corresponding author.

The authors apologize for this error and state that this does not change the scientific conclusions of the article in any way. The original article has been updated.

